# Tensile, Quasistatic and Dynamic Fracture Properties of Nano-Al_2_O_3_-Modified Epoxy Resin

**DOI:** 10.3390/ma11060905

**Published:** 2018-05-28

**Authors:** Zhiwei Duan, Hailing He, Wenyan Liang, Zhenqing Wang, Liang He, Xiaohong Zhang

**Affiliations:** 1 Smart Structrues and Advanced Composite Materials Lab, Harbin Engineering University, Harbin, 150001, China; duanzhiwei@hrbeu.edu.cn (Z.D.); hehailing@hrbeu.edu.cn (H.H.); wangzhenqing@hrbeu.edu.cn (Z.W.); heliang@hrbeu.edu.cn (L.H.); 2 School of Material Science and Chemical Engineering, Harbin Engineering University, Harbin 150001, China; zhangxiaohong@hrbeu.edu.cn

**Keywords:** epoxy resin, nanoparticles, dynamic fracture, 2D-DIC, SHPB

## Abstract

Epoxy resin, modified with different particle sizes (50 nm, 100 nm, 200 nm) and contents (1 wt %, 3 wt %, 5 wt %, 7 wt %) was manufactured. The mechanical behaviors of tensile, quasistatic fracture and dynamic fracture under SHPB (split Hopkinson pressure bar) loading were investigated. The dynamic fracture behaviors of the composites were evaluated by 2D-DIC (digital image correlation) and the strain gauge technique, and the fracture surface was examined by SEM (scanning electron microscope). According to the results, the tensile modulus and strength significantly increased for epoxy resin modified with 5 wt % Al_2_O_3_ of 50 nm. The quasistatic fracture toughness of modified epoxy resin increased with the particle content. However, the fracture toughness of epoxy resin modified with high content fillers decreased for particle agglomeration that existed in epoxy resin. The crack propagation velocity can be decreased for epoxy resin modified with particles under dynamic loading. The dynamic initiation fracture toughness of modified epoxy resin increases with both particle size and content, but when the fillers have a high content, the particle size effects are weak. For the composite under dynamic loading conditions, the toughening mechanism is also affected by particle size.

## 1. Introduction

Particulate-filled epoxy resin composites have been widely used in the engineering field due to their excellent mechanical properties and strong designability, which are useful for different applications. With the development of nanotechnology, nanoparticle-filled composites are attracting increasing attention, to further enhance the mechanical performance of composites. However, the fracture behavior of composites under different loading conditions must be taken into consideration in designing and evaluating mechanical properties.

Epoxy resin is normally modified by adding rigid and soft particles such as rubber, glass, and silica, to improve fracture toughness under impact loading [1,2,3]. Guo et al. [4] and Huang [5] proposed a set of analytical and experimental methods to investigate the crack propagation and fracture behavior of nanostructures. They found an important phenomenon: the interaction between the interfaces and crack may affect the fracture behavior significantly. Nakamura et al. [6,7,8] studied the impact properties of epoxy resin reinforced by silica particles that differ in shape and range in size from 2 to 47 µm. They found that as the particle size decreased, the impact absorbed energy increased for the epoxy resin filled with irregular-, spherical-, and angular-shaped silica particles. However, the agglomeration of particles in resin makes it difficult to strictly distinguish the effect of shape and size. Day [9] used three types of particles to toughen the epoxy resin, and the results indicated that when periphery diameter of the rubbery layer of the particle was over 0.35 µm and the size of the glassy polymer core increased, the toughness of the composite decreased. Evora et al. [10] have studied the effects of nanosize particles of TiO_2_ (with an average diameter of 36 nm) on nanocomposite bulk mechanical properties and dynamic fracture toughness, where an increase in dynamic fracture toughness relative to quasistatic fracture toughness was observed. Zhang et al. [11] added 44 nm nano-CaCO_3_ into isotactic polypropylene (PP), and found that the composite tensile properties did not significantly change but that the notched Izod impact energy of the composites increased significantly. Marur et al. [12] added different alumina particles (50 nm, 500 nm, 5 µm) into the epoxy resin, evaluating static and dynamic fracture toughness (tested via a drop weight tower). They found that particle size significantly affects static and dynamic fracture toughness. Kitey [13] showed that filler–matrix adhesion strength and particles size play an important role in the dynamic fracture behavior of glass-filled epoxy (spherical particles of size 7–200 μm). Under dynamic loading conditions, the strong filler–matrix adhesion has a negative effect on the fracture toughness, while particle size had little or no effect on fracture toughness of strongly bonded specimens, and the particle size effects were relatively more prominent in weakly bonded ones. Jajam et al. [14] found that, compared with the microfiller, the nanofiller did not exhibit better toughening behavior for epoxy resin under the dynamic fracture test. Wetzel et al. [15] studied epoxy resin containing varying amounts of nano-TiO_2_ and nano-Al_2_O_3_, where the quasistatic fracture toughness and flexural strength were improved with increasing nanoparticle contents, and where the fracture toughness was higher for composites modified with small-sized particles. Zamanian [16] modified epoxy resin via the addition of nanosilica with different sizes (14 nm, 20 nm, and 40 nm) and contents (1–10.5 wt %). The tensile modulus and fracture toughness varied with particle size and content, and the epoxy resin modified with small size and high content nanoparticles showed better mechanical properties.

Many studies have discussed epoxy resin modified with different particle sizes and amounts. While 100 nm was the critical dimension, we usually used to discriminate the nanoparticles, and most researchers have not studied the particle size near the critical dimension. Therefore, particles of 50 nm, 100 nm, and 200 nm will be a better choice for evaluating the particle size effect on the fracture behavior, as well as the particle size effect on the toughening mechanisms of the composite under dynamic loading conditions, both of which need to be studied further. Regarding the analysis methods for the fracture behavior of nonhomogeneous composites, the interaction energy integral method [17,18,19,20] can be applied. Guo et al. [17,18,19] proposed a modified domain-independent interaction energy integral, where the interface or boundary effects were taken into account during the simulation processing. Consequently, their methods can be effectively applied to crack propagation problems of the particulate-filled epoxy resin composites with complex interfaces.

In this work, we investigated the mechanical properties of epoxy resin modified with different nanoparticle sizes and contents under quasistatic and SHPB loading conditions. A 2D-DIC (digital image correlation) method and strain gauge technique were used to study the composites dynamic fracture behavior. The fracture morphology was examined by SEM (scanning electron microscope), and the toughening mechanism has been given.

## 2. Materials and Sample Preparation

### 2.1. Materials

Bisphenol-A epoxy resin E51 (Guangzhou Shixian Chemical Industry Co., Guangzhou, China) was used as the matrix. *o*-Phthalic anhydride and tris(2-hydroxyethyl)amine (Tianjin Kemiou Chemical Reagent Co., Tianjin, China) were used as the curing agent and accelerating agent, respectively. Nano-alumina particles with an average diameter of 50 nm, 100 nm, and 200 nm were provided by Shenzhen Crystal Chemical Co., Shenzhen, China.

### 2.2. Samples Preparation

**Figure 1:**
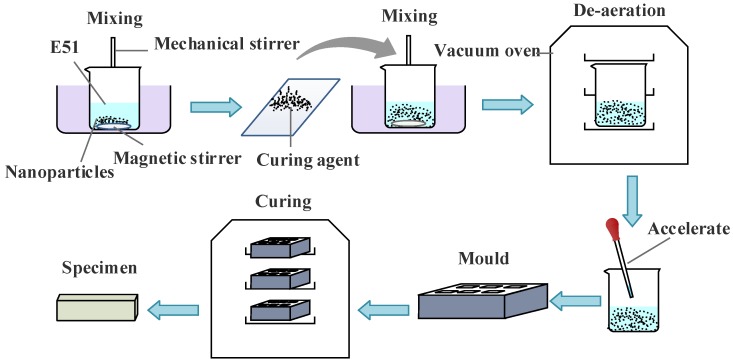
The process of specimens manufacturing.

The nano-alumina particles (50 nm, 100 nm, and 200 nm) were dried for 24 h at 100 °C in order to remove surface water. The epoxy resin was first heated at 80 °C, to reduce the viscosity of the resin and improve the fluidity of the epoxy resin. Then, three sizes of nanoparticles with 1 wt %, 3 wt %, 5 wt %, and 7 wt % were added to the epoxy resin, and the particles were dispersed via a mechanical agitator and magnetic stirring apparatus for 3 h at 60 °C. After that, a curing agent was added into the mixture and stirred until it dissolved completely. In order to remove the air bladder, we put the mixture in a vacuum drying oven, with a vacuum degree of 0.2 KPa for 1 h at 60 °C. After that, the accelerating agent was added, and the mixture was poured into a preheated mold. The curing temperature was set to 120 °C for 30 min, and to 135 °C for 4 h. The whole manufacturing process is shown in Figure 1.

## 3. Experimental Section

### 3.1. Tensile and Quasisstatic Mode-I Fracture Toughness Test

The tensile test was carried out on a Zwick/Roell Z010 Universal Testing Machine (Zwick/Roell, Ulm, Germany) with a capacity of 10 kN at room temperature and with a crosshead speed of 2 mm/min. For the fracture toughness testing, specimens were prepared as a single edge notched bending (SENB) configuration, with dimensions of 106 mm × 20 mm × 10 mm (length × width × thickness). The average length of the pre-crack was 10 mm, which was determined by tapping a new razor blade in the specimens’ notch. Quasistatic mode-I fracture toughness was estimated though a three-point bending test at room temperature with a crosshead speed of 1 mm/min. The fracture toughness of materials is usually expressed by the critical stress intensity factor *K_IC_*, which is defined by Equation (1):

(1) $$K_{IC}=\frac{SP_{c}}{BW^{3/2}}\cdot f(\xi ),$$

(2) $$f(\xi )=\frac{3\xi^{1/2}\left(1.99-\xi \left(1-\xi \right)\left(2.15-3.93\xi +2.7\xi^{2}\right)\right)}{2\left(1+2\xi \right){\left(1-\xi \right)}^{3/2}},$$

where $\xi =\frac{a}{W}$, *P* is the load when cracks begin to propagate, *S* is the span length (*S* = 80), *B* is the specimen thickness, *W* is the specimen width, and *a* is the initial crack length of the specimen.

### 3.2. SHPB Loading System

**Figure 2:**
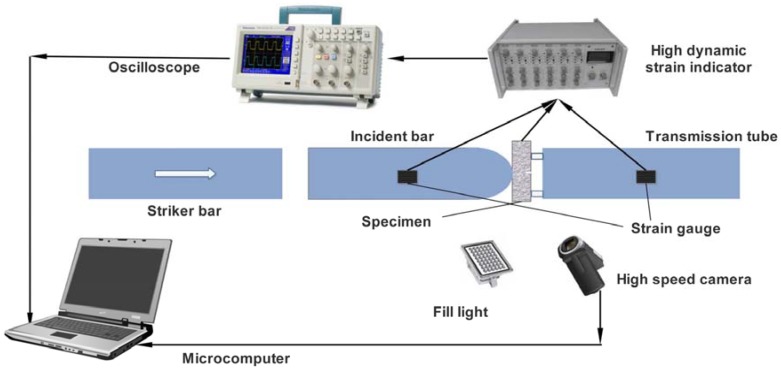
System of split Hopkinson pressure bar (SHPB) loading and data acquisition.

The SHPB test system is shown in Figure 2. The diameter of the striker bar and incident bar were 14.5 mm, and the transmission tube was a hollow pipe with a diameter of 40 mm (inside diameter) and 60 mm (outside diameter). We used a high-speed camera (Phantom V12.1 Camera, Vision Research, Wayne, NJ, USA) to record the deformed information of specimens during the fracture process. According to the recorded information, the displacement fields and strain fields at various times could be calculated by the 2D-DIC (digital image correlation) method [21]. The striker bar speed was set at 7.8 m/s.

### 3.3. Data Analysis

**Figure 3:**
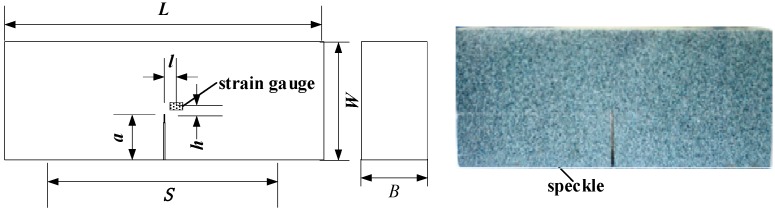
Geometric size of sample (*L* = 64 mm, *S* = 60 mm, *W* = 28 mm, *B* = 14 mm, *a* = 7 mm, *l* = 2 mm, *h* = 2 mm).

For the dynamic fracture toughness test, single edge notched bending specimens with pre-cracks were used. Meanwhile, speckle was sprayed onto the specimen surface and the strain gage was pasted onto the specimen surface, without speckle, near the crack tip. Information on specimen size, strain gage position, and the test span is shown in Figure 3. 

**Figure 4:**
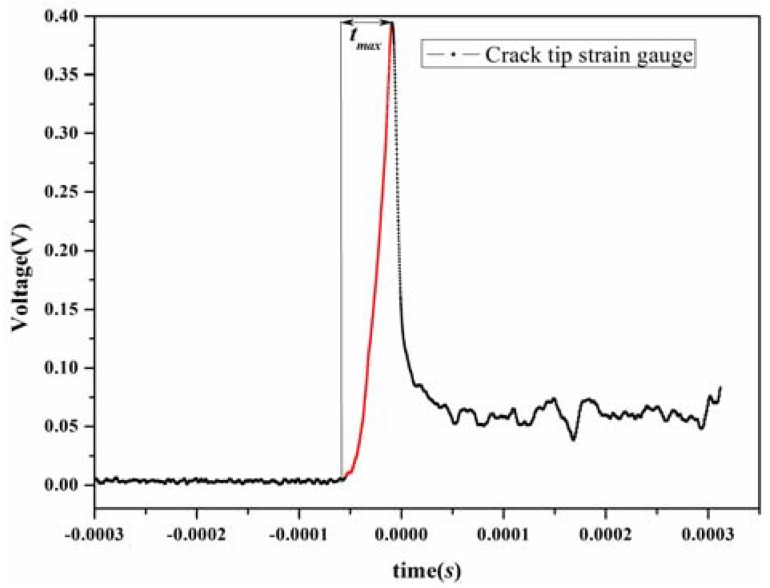
Maximum time of the crack tip strain gauge signal.

The strain gauge was stuck near the crack tip of every test specimen, and the crack initiation time could be evaluated using Equation (3).

(3) $$t_{i}=t_{\max}-\frac{\sqrt{l^{2}+h^{2}}}{c},$$

where *t_max_* was obtained from the strain gauge that was pasted near the crack tip of specimens (during the dynamic fracture test, the signal of the strain gauge near the crack tip is shown in Figure 4), and *c* is the longitudinal wave velocity of epoxy resin, which is measured by ultrasonic measurement (OU1600, Cangzhou Oupu Testing Instrument Company, Cangzhou, China). The velocity of the wave in epoxy resin is 2690 m/s, and *l* and *h* are the horizontal and vertical distances between the strain gauge center and the crack tip, respectively. The dynamic fracture-initiation toughness *K_Id_* (the stress intensity factor (SIF) when the crack begins to grow) is an important parameter to characterize the fracture properties of the composite. Thus, *K_Id_* can be obtained from the value of SIF at the crack growth initiation time, *t_f_*, i.e.,

(4) $$K_{Id}=K_{I}\left(t_{f}\right)$$

The dynamic SIF history can be calculated via the load point displacement, *u_p_(t)* [22], and can be written as

(5) $$K_{I}\left(t\right)=\frac{2}{3}\frac{\beta }{B\sqrt{W}}\frac{u_{p}\left(t\right)}{C\left(\alpha \right)}k_{\beta }\left(\alpha \right),$$

where *u_p_(t)* may be evaluated using Equations (4)–(6) [23],

(6) $$u_{p}(t)=\delta_{I}\left(t\right)-\delta_{II}\left(t\right),$$

(7) $$\delta_{I}\left(t\right)=c_{1}\int_{0}^{t}\left(\varepsilon_{I}-\varepsilon_{R}\right)dt,$$

(8) $$\delta_{II}\left(t\right)=c_{2}\int_{0}^{t}\varepsilon_{T}dt,$$

where $\delta_{I}\left(t\right)$ and $\delta_{II}\left(t\right)$ are, respectively, the displacements of the incident bar and transmission tube. C(α) is the compliance of the specimen with the pre-crack (a more detailed expression has been given by Rubio [22]), and $k_{\beta }\left(\alpha \right)$ is a nondimensional function whose expression can be found in Guinea et al. [24]. During the test, when there is guaranteed to be no contact loss between the incident bar and the specimen, the above equations could be used.

**Figure 5:**
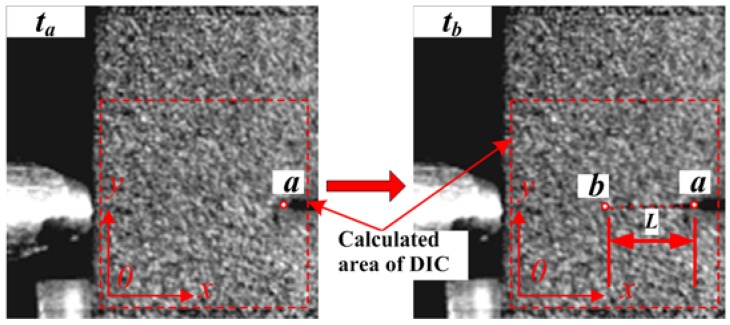
Crack velocity calculated via the DIC method.

The 2D-DIC method was used to study the dynamic fracture behavior by measuring the deformations of the specimens. The crack propagation velocity was estimated from the crack length variation with time, as shown in Figure 5, and it can be evaluated using Equations (9) and (10):

(9) $$L=\sum_{i=1}^{n-1}\sqrt{{\left(x_{i+1}-x_{i}\right)}^{2}+{\left(y_{i+1}-y_{i}\right)}^{2}}*\lambda$$

(10) $$v=\frac{L}{t_{b}-t_{a}}$$

where λ = 0.20477 mm/pixel, and *n* is the numbers of images recorded by the high-speed camera during crack growth. *L* is the crack propagation length for the duration of *t_b_-t_a_*. By calculating the change of time-adjacent images of gray values, we were able to obtain the length of crack growth for a given period. Then, we were able to add up the time-adjacent crack length and the crack growth length *L* from *t_a_* to *t_b_*.

**Figure 6:**
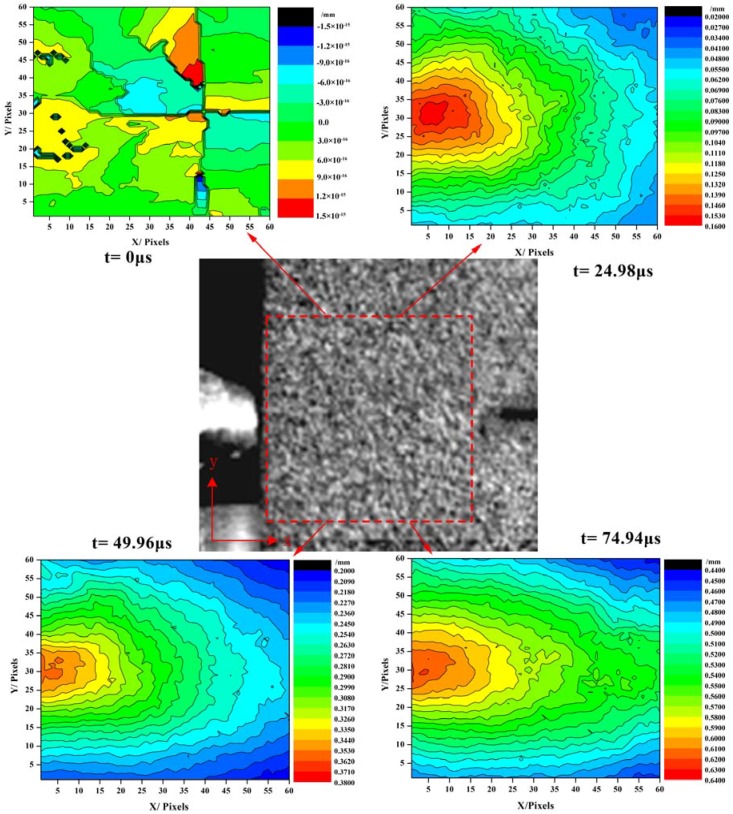
Displacement contour of *u_x_* for *t* = 0 µs, *t* = 24.98 µs, *t* = 49.96 µs, and *t* = 74.94 µs.

**Figure 7:**
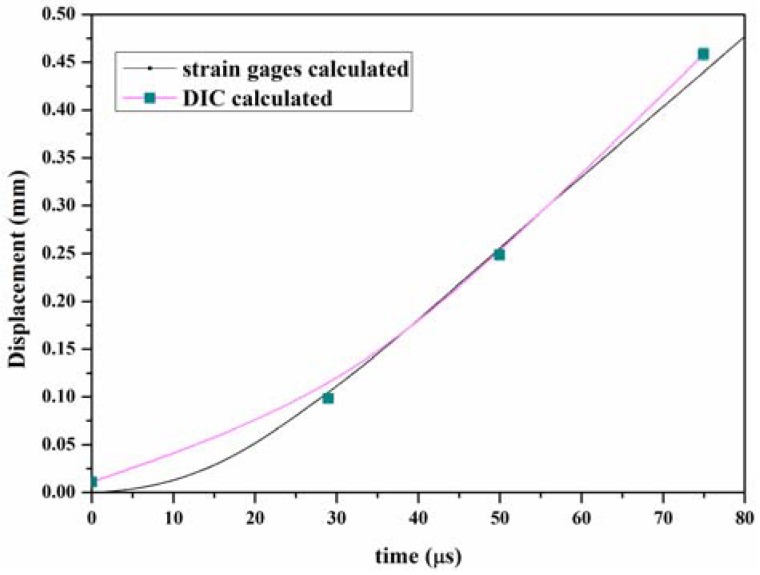
Loading-point displacement evaluated via DIC and Equation (4).

The DIC method was also used to evaluate the load-point (the position where the incident bar contacted the specimen) displacement. A sub-image size of 60 pixels × 60 pixels was chosen to calculate the displacement fields. The displacement fields contours of *u_x_* at *t* = 0 µs (crack begins to grow), *t* = 24.98 µs, *t* = 49.96 µs, and *t* = 74.94 µs are shown in Figure 6. We used the least squares method to estimate the load-point displacement *u_p_(t)* from the *u_x_* displacement field. The load-point displacement *u_p_(t)* obtained by the DIC method and Equation (6), as shown in Figure 7, show very good agreement with the results. Compared with the results obtained from the load-point displacement data, more SIF history results can be obtained via the strain gauge method.

## 4. Results and Discussion

### 4.1. Tensile and Quasistatic Mode-I Fracture Toughness

**Figure 8:**
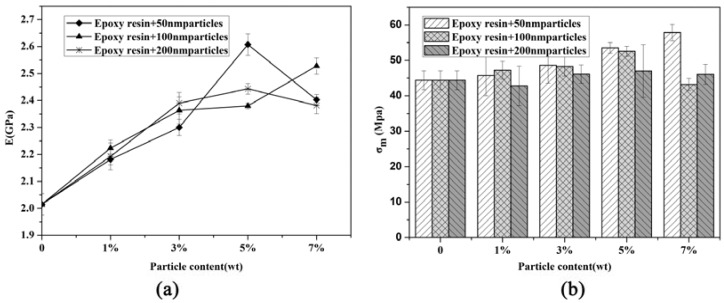
Tensile modulus and strength of epoxy modified with nano-Al_2_O_3_: (**a**) tensile modulus (E), (**b**) tensile strength (σ_m_).

The physical properties of all the particles that modified the epoxy resin are shown in Table 1. The density of the composites was measured by high-precision solid densitometer (ET-220S, ETNALN, Beijing, China). As seen from Table 1, the composite density increased with the particle content, while the particle size had almost no effect on the density. The tensile modulus and strength of epoxy resin modified with different particle sizes and contents is shown in Figure 8, and the modulus data is tabulated in Table 1. A tensile modulus of 2.02 GPa was measured for the unmodified epoxy resin, and a maximum modulus of 2.61 GPa was measured for the epoxy resin modified with 5 wt % of 50 nm particles; an increase of 29.2% was noted compared to that of the unmodified epoxy resin. Meanwhile, for epoxy resin modified with 50 nm and 200 nm nano-Al_2_O_3_ at a high content, the trend was reversed, and the modulus decreased. This variation trend for nanoparticle-modified epoxy resin at a high content was also reported in [14]. Furthermore, the presence of particle agglomerations reduced the effective interface areas, which led to the modulus being lower than expected. When comparing the composites’ elastic modulus of different modified particle sizes, the size effect on the modulus was not obvious for the particles at low content. Therefore, the elastic modulus presented quite contrasting features for different particle content-modified epoxy resins; for composites modified with 50 nm particles, the modulus was higher than other modified epoxy resin. The tensile strength increased with the particles’ content for epoxy resin modified with 50 nm particles, while for epoxy resin modified with 100 nm and 200 nm particles, the nanoparticles’ particle content had little influence on the tensile strength.

**Figure 9:**
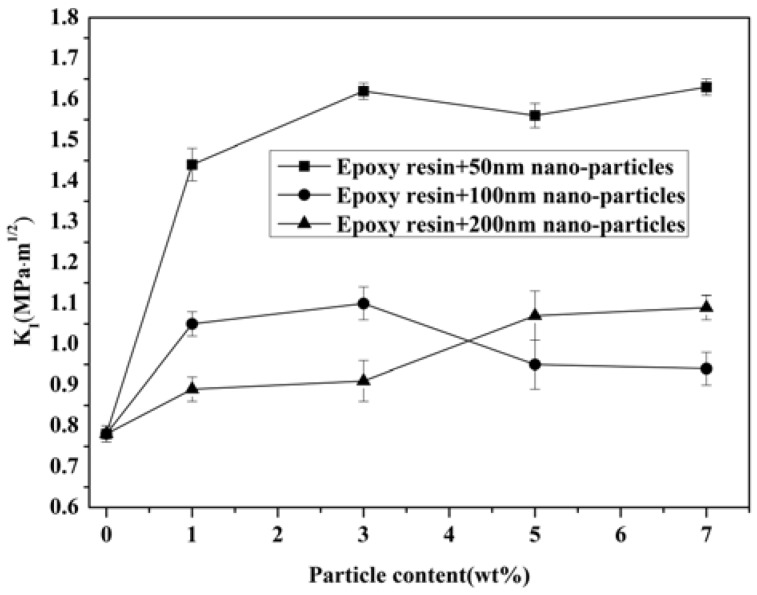
Mode-I fracture toughness of epoxy resin modified by nano-Al_2_O_3_.

**Figure 10:**
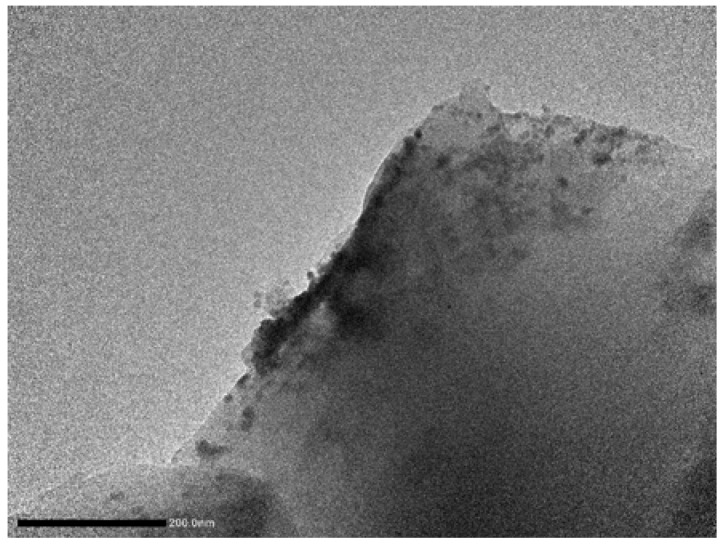
Agglomeration on the fracture surface of epoxy resin modified with 7 wt % of 50 nm.

Figure 9 shows the mode-I fracture toughness calculated by Equation (1), *K_IC_*, for both unmodified and modified epoxy resin. In the case of the neat epoxy resin, the fracture toughness *K_IC_* was determined to be 0.78 MPa·m^1/2^, while a maximum *K_IC_* of 1.56 MPa·m^1/2^ was measured for epoxy resin with 7 wt % of 50 nm. When comparing the toughness of modified epoxy resin with different particle sizes, epoxy resin modified with 50 nm particles showed higher values. The fracture toughness of 50 nm particle-modified resin is higher than when 200 nm nano-Al_2_O_3_ particles were incorporated into the matrix. Meanwhile, the toughness of *K_IC_* decreased for epoxy resin modified with 5 wt % of 50 nm and 100 nm particles. For epoxy resin modified with high particle contents, the increasing trend reversed, which suggests the occurrence of nano-Al_2_O_3_ agglomeration. The fracture surface morphology of epoxy resin modified with 7 wt % of 50 nm particles, examined by TEM, shows the occurrence of agglomeration (Figure 10). The occurrence of agglomeration means a reduction in numbers of effective particles in epoxy resin; therefore, the fracture toughness of modified epoxy resin was reduced.

### 4.2. Effect of Particle Size and Content on the Crack Propagation Velocity

The crack velocity (steady state crack velocity) was estimated from Equation (10). The velocities of the neat epoxy resin modified with nano-Al_2_O_3_ with different sizes (50 nm, 100 nm, 200 nm) and contents (1 wt %, 3 wt %, 5 wt %, 7 wt %) under dynamic loading are shown in Table 2. The highest crack velocity was 286.91 m/s, for the neat epoxy resin. When comparing the crack velocity with that of the modified resin, the following could be found: through the incorporation of particles into epoxy resin, the crack velocity could be effectively reduced. However, for epoxy resin modified with 50 nm Al_2_O_3_, the crack velocity did not obviously decrease, and the maximum velocity reduction rate (compared with the neat epoxy resin) was 14.29%. For epoxy resin modified with 100 nm and 200 nm particles, the maximum velocity reduction rates were 36.95% and 37.96%, respectively. Thus, for modified epoxy resin under dynamic loading, the crack velocity could be reduced more effectively by larger particle sizes. The results in ref [25] showed that the crack propagated at a significantly higher velocity in nanocomposites than it did in composites modified with micron-sized fillers. Under dynamic loading conditions, the crack path was more easily deflected for epoxy resin modified with larger-sized particles than when modified with smaller-sized particles. Meanwhile, the crack propagation velocity can be reduced with an increase in particle content.

### 4.3. Effect of Particles Size and Content on the Dynamic SIF

**Figure 11:**
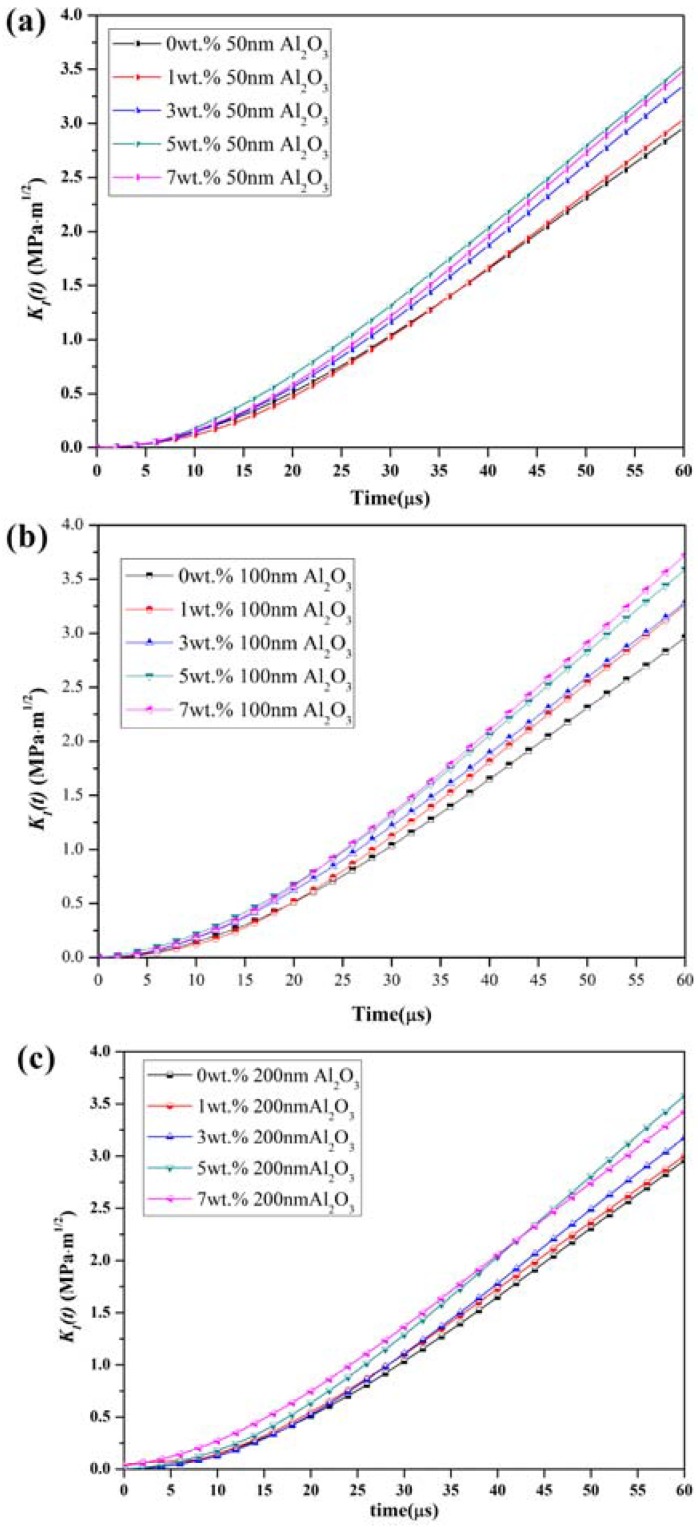
Dynamic stress intensity factor (DSIF) history of composites with different particle size and content: (**a**) epoxy resin modified with 50 nm particles; (**b**) epoxy resin modified with 100 nm particles; (**c**) epoxy resin modified with 200 nm particles.

**Figure 12:**
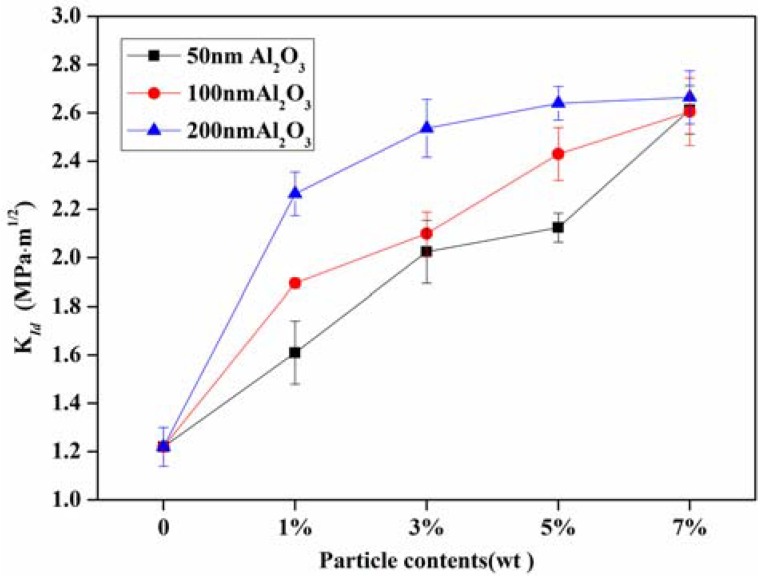
Crack initiation toughness (*K_Id_*) of particle modified epoxy resin.

The dynamic stress intensity factor (DSIF) histories of the epoxy resin modified with different particle sizes and contents were evaluated by Equation (5), as shown in Figure 11. In this plot, the crack initiation time was estimated by Equation (3) and listed in Table 2. Therefore, the dynamic initiation toughness of both unmodified and modified epoxy resin, can be calculated by Equation (4), as shown in Figure 12. For the epoxy resin modified with 50 nm particles at 1 wt %, the *K_I_(t)* history curve was similar. With the particle content increasing, the slope of the *K_I_(t)* history curve also increased, as can be seen in Figure 11a,b. For the epoxy resin modified with 200 nm particles of 1~5 wt %, the slope of the *K_I_(t)* history curve increased with the particle content, while for particle content over 5 wt %, the slope decreased.

Figure 12 shows the dynamic initiation fracture toughness of the unmodified and modified composite: the particle size had a significant influence on the initiation fracture toughness. The fracture toughness increased by 31.9%, 55.73%, and 86.1% for the epoxy resin with 50 nm, 100 nm, and 200 nm particles modified at 1 wt %, respectively. Meanwhile, the initiation fracture toughness increased with the particle content. Notably, the dynamic fracture toughness was almost equal for the epoxy resin modified with different sizes particles at 7 wt %. The reason for this is that the more particles incorporated into the epoxy resin, the more interfaces there were in the composites, especially for epoxy resin modified with small-sized particles. Therefore, for a high content modified filler, the particle size effects can be neglected, and more energy will be expended during the fracture process. The toughening mechanism of particle sizes will be discussed in Section 4.3.

**Figure 13:**
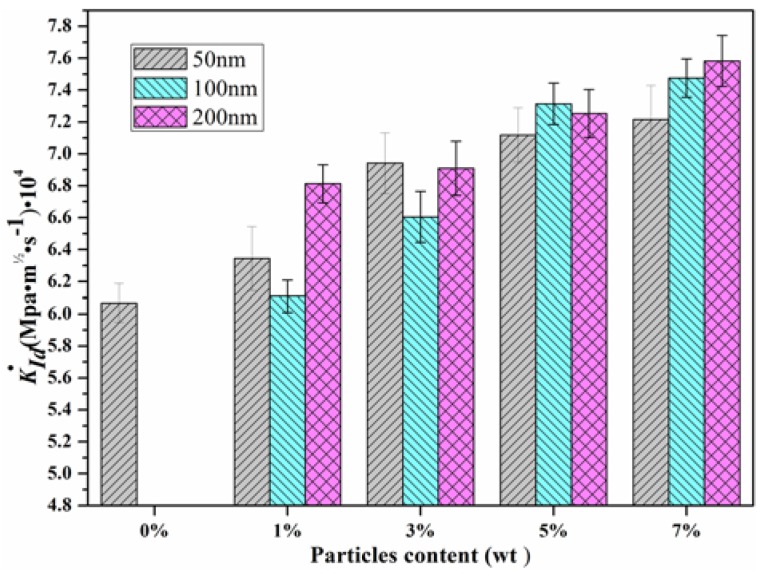
Loading rate of unmodified and modified epoxy resin (${\dot{K}}_{Id}=dK_{Id}/dt$).

Table 2 shows the crack initiation time of unmodified and modified epoxy resin. In Table 2, the crack initiation time of the unmodified epoxy resin is 33.1 μs, and for modified epoxy resin, it increased with the particle content. This is an indication that with more particles added into epoxy resin, the crack initiation time will be delayed. The loading rate of a cracked specimen under dynamic loading condition is defined as ${\dot{K}}_{Id}=dK_{Id}/dt$. Therefore, an obvious question to pose is whether the epoxy resins modified with different particle sizes and contents are rate dependent? It is therefore necessary to compare the loading rate of unmodified and modified epoxy resins. The loading rate of unmodified and modified epoxy resins is shown in Figure 13. We found that the particle content could affect the loading rate; for epoxy resin modified with low content particles, the size effect was obvious; there was no significant size effect for epoxy resin modified with high content particles.

### 4.4. Toughenning Mechanisms

**Figure 14:**
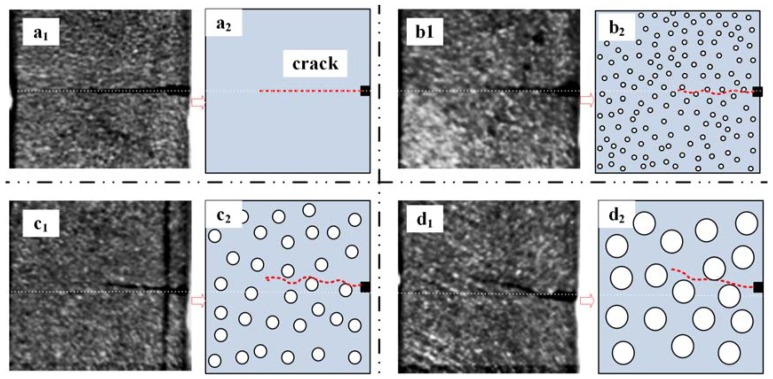
The mechanism of particle size effect on the dynamic fracture toughness: (**a_1_**,**a_2_**) neat epoxy; (**b_1_**,**b_2_**) 50 nm Al_2_O_3_-filled epoxy (1 wt %); (**c_1_**,**c_2_**) 100 nm Al_2_O_3_-filled epoxy (1 wt %); (**d_1_**,**d_2_**) 200 nm Al_2_O_3_-filled epoxy (1 wt %).

When comparing the crack propagation velocity, the large-sized particles (at the same content) could effectively reduce the velocity. The energy dissipation mechanisms of the particle size effect on the composite are shown in Figure 14 (Figure 14a_1_–d_1_ represents the crack propagation of the neat epoxy resin, and the epoxy resin modified with 50 nm (1 wt %), 100 nm (1 wt %), and 200 nm (1 wt %) at the same time during the SHPB loading process). Figure 14a_1_,b_1_, shows that the crack propagated almost along a straight line; however, the dynamic initiation fracture toughness was higher than that of neat epoxy resin, as shown in Figure 12. This can be explained by Figure 14a_2_,b_2_. During the crack growth, for the epoxy resin modified with 50 nm particles, the crack will be hampered by particles, and a tiny deflection path will be formed, increasing the dynamic fracture toughness. With the particle size increasing as shown in Figure 14c_1_,d_1,_ the angle of crack deflection became larger. Figure 14c_2_,d_2_ gives the crack path deflection mechanisms of epoxy resin modified with 100 nm particles. With a particle content at 7 wt %, the crack initiation toughness (*K_Id_*) of the composite tended to be equal. The reason for this is that the number of particles was higher for 50 nm than for either 100 nm or 200 nm; particle agglomeration of 50 nm particles may lead to the energy necessarily consumed for crack initiation being the same for the epoxy modified with different-sized particles at 7 wt %.

**Figure 15:**
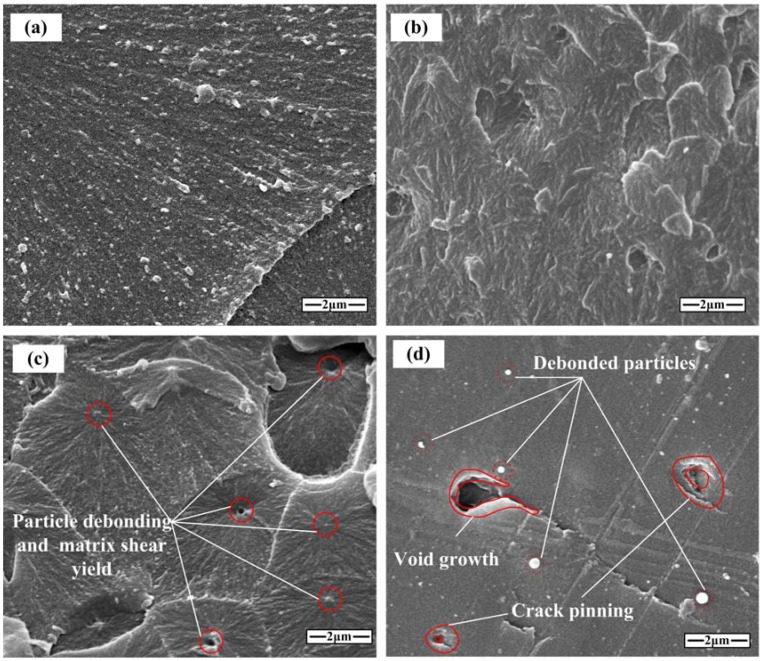
SEM micrographs of the dynamic fracture surface (**a**) neat epoxy; (**b**) 50 nm Al_2_O_3_-filled epoxy (3 wt %); (**c**) 100 nm Al_2_O_3_-filled epoxy (3 wt %); and (**d**) 200 nm Al_2_O_3_-filled epoxy (3 wt %).

It has been suggested that in epoxy resin, crack deflection processes play an important role in toughening, and a theory has been proposed by Faber and Evans [26]. It is assumed that a crack can be deflected at an obstacle and the crack propagation path could be changed by tilting and twisting. However, the toughening effect provided by nano-Al_2_O_3_ explained by the crack deflection theory is not enough. The detailed damage information of the fracture surface observed by visual inspection was not effective, so the fracture surfaces were examined via scanning electron microscopy (SEM) (Hitachi, City, Japan). Figure 15 shows the micrographs of the dynamic fractured surfaces of the neat epoxy resin, and epoxy modified with 50 nm, 100 nm, and 200 nm nano-Al_2_O_3_ at 3 wt %, respectively. For the neat epoxy resin, river-like lines were formed after specimen fracture, as shown in Figure 15a, which reflects the shear plastic deformation [27]. The fracture surface of epoxy resin modified with 50 nm particles shows that a high amount of crazed shear band and plastic deformation occurred on the fracture surface, and that the dynamic fracture toughness increased via the two mechanisms. With particle sizes increasing, some particles that debonded from the matrix can be seen in Figure 15c,d, which reveals that dynamic fracture toughness may be improved by both particle debonding and pullout for the matrix modified with 100 nm and 200 nm particles. Meanwhile, for the epoxy resin modified with 100 nm Al_2_O_3_, a plastic void growth could be observed after particle debonding, and this results in matrix plastic deformation [28,29]. As shown in Figure 15d, the crack pinning existed on the fracture surface for the epoxy resin modified with 200 nm Al_2_O_3_. The crack pinning mechanism has been explained for epoxy resin modified with microparticles [30], but also for nanoparticle-reinforced polymers [27]. In short, by using the nano-Al_2_O_3_-modified epoxy resin, dynamic fracture toughness can be enhanced, and particle size and content could affect the toughening mechanisms differently.

## 5. Conclusions

In this study, the strain gauge method and DIC method were used to calculate the dynamic fracture behavior of epoxy resin modified with different nano-Al_2_O_3_ sizes (50 nm, 100 nm, 200 nm) and contents (1 wt %, 3 wt %, 5 wt %, 7 wt %) under SHPB loading. The tensile and quasistatic mode-I fracture toughness properties of particle-modified epoxy resin have also been given. The influence of particle size and content on dynamic fracture toughness, loading rate, crack propagation velocity (at a steady state), and particle toughness mechanisms were discussed. From the experimental results, we can conclude the following:

◆ The tensile modulus of epoxy resin modified with three Al_2_O_3_ particles of different size increased with particle content, with the highest increase of 29.2% for epoxy resin modified with 5 wt % of 50 nm particles. The tensile strength significantly increased for the epoxy resin modified with 5 wt % and 7 wt % of 50 nm particles, while there were almost no changes for the epoxy resin modified with 100 nm and 200 nm particles with different contents.
◆ The quasistatic fracture toughness *K_IC_* had a maximum value of 1.56 MPa·m^1/2^ for epoxy resin modified with 7 wt % of 50 nm particles, and the *K_IC_* increased with particle content. While the toughness did not increase with particle content for the fillers at high content, the agglomeration reduced the effective number of Al_2_O_3_ particles in the epoxy resin, thus reversing the trend.
◆ Crack propagation velocity was evaluated by the DIC method for the composites under dynamic loading condition; the crack path was more easily deflected for large-sized particles than for small-sized particles. Meanwhile, the crack propagation velocity could be reduced with an increase in particle content.
◆ The dynamic initiation fracture toughness *K_Id_* increased with particle content and size. The dynamic fracture toughness was almost equal for epoxy resin modified with three particle sizes at 7 wt %. For high content fillers, the effect of the particle size could be neglected, and more energy would be expended during the fracture process. The loading rate could be affected by the particle content, while for the epoxy resin modified with low content particles, the effect was obvious; there was no significant size effect for epoxy resin modified with high content particles.
◆ It has been suggested that in particle-modified epoxy resin, the crack deflection processes play an important role in toughening; under dynamic loading and with an increase in particle size, the crack deflection angle became larger. Toughening mechanisms are affected by particle size: for the epoxy resin modified with 50 nm particles, crazed shear band and shear plastic deformation improved the fracture toughness; for the resin modified with 100 nm particles, debonding and plastic void growth were observed; finally, particle debonding and crack pinning were shown to occur in epoxy resin modified with 200 nm particles.

## Figures and Tables

**Table 1:** The basic physical parameter of nano-Al_2_O_3_-filled epoxy resin

Particles Size	Particle Content Nano-Al_2_O_3_ (wt %)	Density *ρ* (g/cm^3^)	Elastic Modulus *E* (GPa)	Poisson’s Ratio *ν*
0	0	1.213 ± 0.004	2.02 ± 0.04	0.36
50 nm	1	1.238 ± 0.003	2.18 ± 0.02	0.35
	3	1.256 ± 0.004	2.30 ± 0.03	0.35
	5	1.247 ± 0.006	2.61 ± 0.04	0.35
	7	1.295 ± 0.001	2.40 ± 0.02	0.35
100 nm	1	1.234 ± 0.005	2.22 ± 0.03	0.35
	3	1.249 ± 0.002	2.36 ± 0.05	0.34
	5	1.272 ± 0.004	2.38 ± 0.01	0.35
	7	1.291 ± 0.003	2.52 ± 0.03	0.36
200 nm	1	1.237 ± 0.001	2.19 ± 0.05	0.35
	3	1.256 ± 0.004	2.39 ± 0.04	0.35
	5	1.275 ± 0.006	2.44 ± 0.02	0.34
	7	1.294 ± 0.003	2.38 ± 0.03	0.35

**Table 2:** Average steady state crack propagation velocity and crack initiation time of nano-Al_2_O_3_-filled epoxy resin.

Particle Size	Content (wt %)	Crack Velocity (m/s)	Crack Initiation Time *t_i_* (μs)
0	0	286.91	33.1
50 nm	1	277.42	38.4
	3	270.51	40.4
	5	245.92	41.0
	7	254.12	49.8
100 nm	1	229.52	41.0
	3	213.13	43.6
	5	192.34	44.9
	7	180.34	42.4
200 nm	1	213.13	50.1
	3	204.93	54.7
	5	188.54	52.9
	7	178.24	56.4
